# Video triage in calls concerning children with fever at an out-of-hours medical helpline: a prospective quality improvement study

**DOI:** 10.1186/s13049-023-01106-9

**Published:** 2023-08-29

**Authors:** Caroline Gren, Asbjoern Boerch Hasselager, Gitte Linderoth, Marianne Sjølin Frederiksen, Fredrik Folke, Annette Kjær Ersbøll, Hejdi Gamst-Jensen, Dina Cortes

**Affiliations:** 1 Department of Pediatrics and Adolescence Medicine, Copenhagen University Hospital - Amager and Hvidovre, Copenhagen, Denmark; 2 Department of Clinical Medicine, Faculty of Health and Medical Sciences, University of Copenhagen, Copenhagen, Denmark; 3 Department of Pediatrics and Adolescence Medicine, Copenhagen University Hospital - Herlev and Gentofte, Copenhagen, Denmark; 4 Department of Anesthesia and Intensive Care, Copenhagen University Hospital - Bispebjerg and Frederiksberg, Copenhagen, Denmark; 5 Copenhagen University Hospital - Copenhagen Emergency Medical Services, Copenhagen, Denmark; 6 Department of Cardiology, Copenhagen University Hospital - Herlev and Gentofte, Copenhagen, Denmark; 7 National Institute of Public Health, University of Southern Denmark, Copenhagen, Denmark; 8 Department of Anesthesia, Center of Head and Orthopedics, Copenhagen University Hospital Rigshospitalet, Copenhagen, Denmark

**Keywords:** Triage, Telemedicine, Primary health care, Fever, Pediatrics, Telenursing, Parents, Patient participation

## Abstract

**Background:**

Parents often contact out-of-hours services due to worry concerning febrile children, despite the children rarely being severely ill. As telephone triage of children is challenging, many children are referred to hospital assessment. This study investigated if video triage resulted in more children staying at home. Secondary aims included safety, acceptability and feasibility of this new triage tool.

**Methods:**

In this prospective quality improvement study, nurse call-handlers enrolled febrile children aged 3 months-5 years to video or telephone triage (1:1), with follow-up within 48 h after call. The setting was an out-of-hours call-center for non-urgent illness in Copenhagen, Denmark, receiving over 1 million calls annually and predominately staffed by registered nurses. Main outcome measure was difference in number of children assessed at hospital within 8 h after call between video-and telephone triage group. Rates of feasibility, acceptability and safety (death, lasting means, transfer to intensive care unit) were compared between the triage groups.

**Results:**

There was no difference in triage outcome (home care vs. hospital referral) or number of patients assessed at hospital between triage groups. However, more video triaged patients received in-hospital treatment, testing and hospitalization.

**Conclusion:**

Video triage was feasible to conduct, acceptable to parents and as safe as telephone triage. The study did not show that more children stayed at home after video triage, possibly because the allocation strategy was not upheld, as video triage sometimes was chosen in cases of complex and severe symptoms, and this likely has changed study outcome.

*Trial registration*: Clinicaltrials.gov.: Id NCT04074239. Registered 2019-08-30. https://clinicaltrials.gov/ct2/show/study/NCT04074239

## Introduction

Telephone triage is used extensively during out-of-hours (OOH) healthcare and is a crucial tool in prioritizing both pre-hospital and in-hospital resources. Telephone triage is a challenging task, and children, the relatively most frequent group of callers, are inherently difficult to triage. This is mainly due to second-hand consultations via the parents, and the paucity of visual input, which complicates the assessment of the frequently unspecific symptoms in children [1–4]. Fever is one of the most common reasons for contacting primary- and OOH care in the pediatric population [5, 6]. Fever causes parents to worry, and worry is a main trigger of contact concerning children to OOH care [7–9]. High degree-of-worry (DOW) was associated with face-to-face assessment at hospitals in a study of an OOH general population of callers at the Medical Helpline 1813 (MH1813) in Copenhagen, Denmark [10], as well as in a mixed pediatric population at MH1813 [9].

Furthermore, many parents have misconceptions about fever, such as dangerous side effects, measurement methods, the definition of fever, and how to manage it [11, 12]. Despite this worry, fever is rarely a sign of severe illness, but children’s general state and accompanying symptoms may be complex and challenging to assess. It is difficult to find data of the diagnoses of children with acute illness contacting an OOH call-center, but some data from other healthcare systems exist; only about 1% or less of children with acute illness were seriously ill in Belgian primary care [13] and US emergency departments [14], and only 7% of almost 16,000 febrile children aged less than five years in an Australian pediatric emergency department had a serious infection [15]. As such, it is not easy to find the few severely ill children in a busy OOH call-center.

Moreover, several studies have shown that non-verbal impressions such as tone of voice, breathing and pauses are a crucial part of call-handling [2, 16] and that dealing with second-hand information is experienced as difficult among call-handling nurses [1, 3]. In nearly half of the complaints regarding calls to emergency medical services in Sweden, the call-handler did not speak directly to the ill person [17]. The absence of visual assessment in call-handling is also a relevant issue considering that clinicians’ clinical judgement based on their impression of the patient is a very important factor in identifying severely ill children in primary care [18, 19]. Also, a febrile young child or infant being ‘well-appearing’, is used as an important marker of how intensive testing and treatment needs to be [20, 21]. Lastly, parents often expect a physical examination when seeking OOH care with an acutely ill child, especially if concerning fever [7, 22].

This present study investigated live video streaming (video triage) of febrile children at an OOH call-center. Previously, a study reported that video triage of young children with respiratory symptoms at the same call-center, was safe, feasible and acceptable to both parents and call-handlers [23]. The users’ experiences of video triage in both this present study and the study on respiratory symptoms were very positive: the service was highly appreciated by both parents and call-handlers, and call-handlers’ reassurance about triage decision was higher when having used video, as were parents’ general satisfaction and reassurance about assessment [24].

This present study investigated if the new triage tool video triage resulted in a higher proportion of patients staying at home the next 8 h after the call, while also identifying the most ill children and referring them to hospital. The study also investigated safety, feasibility and acceptability of video triage.

Other secondary aims included if video triage reduced the parents’ DOW more than standard telephone triage, and if parental DOW was associated with the child being assessed at hospital. The study investigated parental DOW before and after call, and call-handlers’ DOW after the call. Moreover, secondary aims included an investigation of a possible reduction of the parents’ DOW when using video triage, and if the parental DOW was associated with the child being assessed at a hospital. As parental worry often is the reason for contacting healthcare services, it could potentially be of great interest if video triage could decrease parents’ worry, to prevent renewed contact and need of referral to hospital assessment.

## Methods

### Setting

The study was conducted at the OOH Medical Helpline 1813 (MH1813) in Copenhagen, Denmark, which is open 24/7 for injuries and OOH for medical illness. An assessment at a hospital OOH in Denmark requires referral from either the Emergency Medical Services or an OOH service such as MH1813. Self-referral is largely discouraged. MH1813 provides service for a catchment population of 1.8 million citizens and is predominately staffed by registered nurses and supplemented by physicians who both answer calls and supervise. The call-handlers basically have two options: (1) telephone consultation with advice on self-care, possibly with a recommendation to contact their general practitioner (GP) the next workday (or renewed contact to MH1813 if needed), or (2) hospital referral, either to a low-acuity urgent care clinic, or to an emergency department, reflecting different grades of perceived urgency. Calls are forwarded to the ambulance services in the event of potentially life-threatening situations. MH1813 receives over 1 million calls annually of which 25% concern children under the age of 12 years [25]. The service is free-of-charge and paid through taxes.

### Design

A prospective quality improvement study design was used, where a group of experienced nurse call-handlers invited parents of young children to participate. In all calls matching the inclusion criteria and where the parent consented to participation, the call-handlers were instructed to perform standard telephone triage in one call and video triage in the next call and so on, to get two comparable groups. In that way, the two groups would optimally only differ by triage method. After all included calls, the call-handlers filled out an electronic questionnaire about the call and their experiences and sent a questionnaire to the participating parents by text message. The study period lasted from September 1, 2019, to January 31, 2020. Children aged 3 months to 5 years with fever were eligible for inclusion. It was not mandatory that the parents had measured the temperature, even though they were encouraged to do so by the call-handlers. Exclusion criteria were foreign telephone number, previous participation in the project or neither Danish- nor English-speaking parent. The present study used a similar set-up as the previously described video triage study concerning respiratory symptoms [23].

Moreover, the study also included the parents’ DOW before and after having talked to the call-handler, and both scores were reported in questionnaires *after* they had talked to the call-handler. The questionnaire was sent to the parent’s smartphone directly after the call. The parents were asked “BEFORE (*or AFTER*) you had talked to the nurse at the helpline 1813, how worried were you about the situation that you called about, on a scale from 1 to 5, where 1 is minimally worried and 5 is maximally worried?”.

**Fig. 1:**
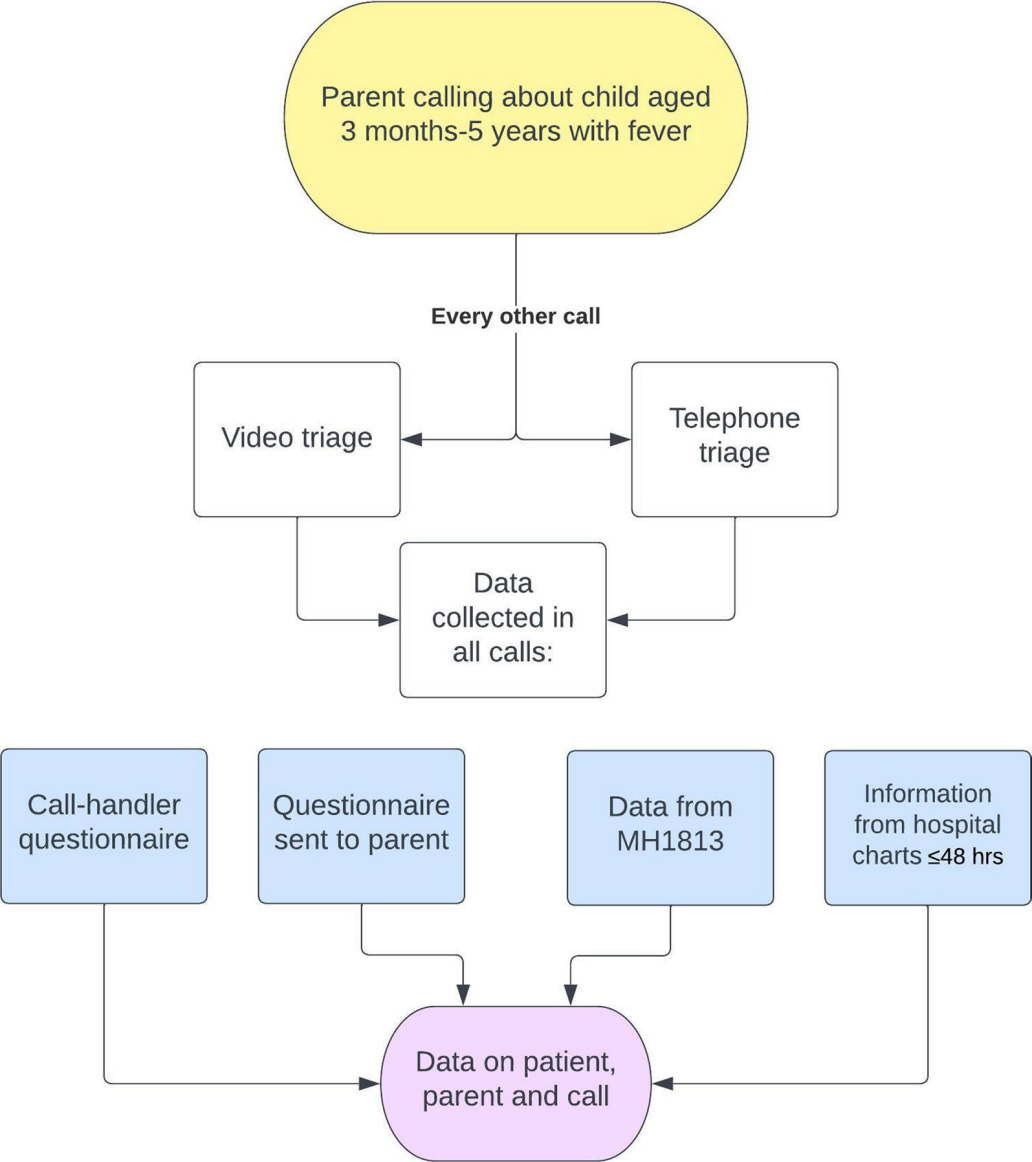
Study design

In addition, the call-handlers rated their DOW of the child immediately after each call. DOW was registered in the survey question “On a scale from 1 to 5, where 1 is minimally worried and 5 is maximally worried, how worried are you that the child is seriously ill?”. The design of the study is illustrated in Fig. 1.

The remaining survey responses of both call-handlers and parents and the actual surveys can be found in a previous article [24], and a more thorough explanation of the concept of degree-of-worry has previously been published [26].

The technological set-up for video streaming was provided by GoodSAM: Instant-on-Scene (https://www.goodsamapp.org/). It has the advantage of being browser based, so the parents did not need to install an application. The call-handler sent a text message with an activation link from the GoodSAM web page, and when the parent gave consent, the video camera opened and the parent streamed the video footage to the call-center. The details regarding the technology are described elsewhere [27].

### Outcome measures

Outcome data were derived from four sources: MH1813 patient records, questionnaire responses from parents and call-handlers, and the hospitals’ patient charts. The primary outcome of the study was to investigate if video triage could result in 10-percentage points more patients being able to stay at home during the first 8 h after the call. In 2018 56% children were triaged to stay at home. The time frame, the first 8 h after the call, was chosen to decrease the risk of including a natural deterioration that had nothing to do with the call and triage decision when including the patient in the study. If a longer time frame had been chosen, there’s a risk that a natural worsening could be interpreted as a wrongful triage decision at time of inclusion.

The patients’ age, gender and triage outcome were retrieved from MH1813 patient records, and reason for contact was retrieved from the call-handlers’ questionnaire. Triage outcome was primarily divided into (1) the patient being advised to stay at home, possibly with contact to GP or MH1813 if necessary, or (2) into hospital referral. It was further registered if the children referred to hospital were referred to a pediatric emergency department (PED) or to a pediatric urgency care clinic (PUCC). This reflects the urgency perceived by the call-handler, as only suspected minor illnesses are to be assessed at PUCCs, where no possibility for treatment or extensive paraclinical testing exists. All included patients were followed-up in the region’s electronic hospital charts up to 48 h after the call. If a patient had been assessed at a hospital, the chart was read to identify time, location, International Statistical Classification of Diseases and Related Health Problems (ICD-10) diagnosis, treatment, and duration of the child’s stay at the hospital. ICD-10 diagnoses were gathered in groups of similar character. Adverse events, defined as transfer to intensive care unit, signs of lasting means or death were also identified in the hospitals’ patient charts. The follow-up was carried out 2–8 days after the call.

The parental DOW was investigated before and after calls in the two study groups. It was studied if video triage reduced the parents’ DOW, and if the parental DOW was associated with the child being assessed at a hospital, both in total and in the two separate study groups. It was also investigated if DOW was associated to triage group.

Safety was defined as the occurrence of adverse events (death, signs of lasting means or transfer to intensive care unit) and feasibility as the rate of successful video calls, and acceptability as how many parents that consented to participate in video triage.

### Statistical analysis

Patients’ baseline characteristics (age, gender, triage response and symptom) were described with frequency (number, percentage), median and interquartile range (IQR). Differences in triage response, symptoms registered by call-handler and hospital outcome between the video and telephone triage groups were analyzed using chi square-test, Mann–Whitney test, logistic regression or multinomial logistic regression as appropriate. Logistic regression and multinomial logistic regression models were presented as odds ratio (OR) estimated with corresponding 95% confidence intervals (95% CI) and were also used for analyzing the association between DOW and hospital assessment.

DOW-scorings were grouped in low (1 + 2) medium (3) and high (4 + 5) in the regression analyses, due to few observations in some categories. As DOW-scores were not normally distributed, they were presented as median with corresponding IQR (interquartile range). Differences in DOW-scores between groups were tested with Kruskal Wallis-test. Association between DOW-score and triage mode was studied using Fisher’s exact test. A 95% CI for proportions was calculated by Wilson Binominal Proportion Confidence Interval. P values less than 0.05 in two-sided tests were considered statistically significant. The statistical analyses were made with SAS Enterprise Guide 7.1 (SAS Institute, Cary, NC, USA), and Open Source Epidemiologic Statistics for Public Health (www.OpenEpi.com).

A sample size of 774 children divided into two groups was needed in order to detect a 10-percentage point increase in children triaged to stay at home, from 56% in the general pediatric MH1813-population in 2018 [25], to 66%, with a power of 80%, a two-sided test and a 5% significance level.

### Ethics

The study was a quality improvement study, and the Research Ethics Committee in the Capital Region of Denmark deemed approval was not indicated (Journal number H-19037554), and participant consent was hence not needed. All participating parents were however informed about the study and gave verbal consent. The managements of the hospitals in the Capital Region with pediatric departments or pediatric urgent care clinics approved access to the patient records, as well as the management of Copenhagen Emergency Medical Services.

## Results

### Patient outcome

**Fig. 2:**
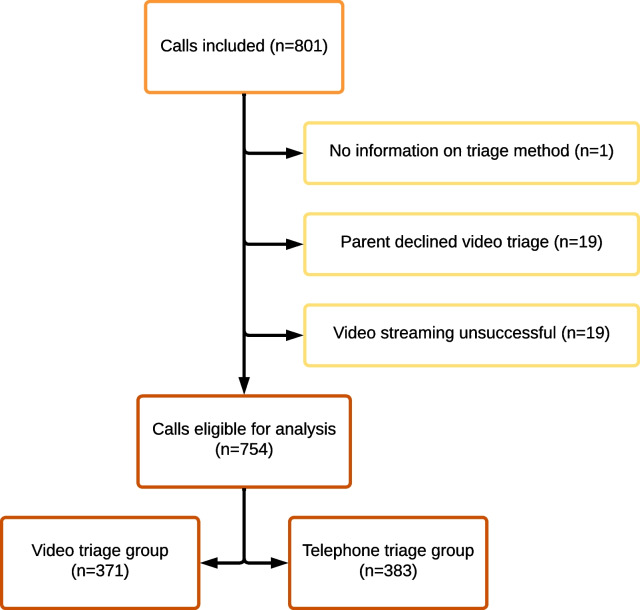
Patient flow

In total, 801 calls were included, and 754 were eligible for analysis, distributed with 371 in the video triage group and 383 in the telephone triage group, Fig. 2. The 19 cases of unsuccessful video streaming were caused by too poor image quality or because the connection between the parent’s telephone and the call-handler’s computer never was achieved. The rate of acceptability was high, as only 19 parents (2.4%) declined to participate.

There was a similar distribution of age and sex between the video and telephone triage groups. The occurrence of different symptoms as registered by the call-handler seemed to vary between the groups for some of the symptoms, for example respiratory symptoms and rash (Table 1a). Furthermore, there was no difference in staying at home between the video triage group and the telephone triage group (53.1% versus 55.6%, OR = 0.90 (95% CI 0.68–1.20)) (Table 1b). Significantly more were referred to a PED in the video triage compared with the telephone triage group (24.8% vs. 13.1%, OR = 2.69 (95% CI 1.73–4.20)).

**Table 1:** Characteristics of included patients in the video and telephone triage group, (a), and outcomes concerning triage outcomes at MH1813 (b)

	Video triage group, n = 371	Telephone triage group, n = 383	OR (95% CI)	p value
(a)				
Age, median (IQR)	1.4 (0.9–2.5)	1.4 (0.9–2.6)		
Male gender, n (%)	216 (58.2%)	213 (55.6%)		
Symptom registered by call-handler, n (%)				
Fever and respiratory symptoms	163 (47.0%)	146 (42.5%)		
Fever without focus	118 (34.0%)	127 (37.0%)		
Fever and other symptoms	31 (8.9%)	47 (13.7%)		
Fever and vomiting/diarrhea	17 (4.9%)	10 (2.9%)		
Fever and rash	9 (2.6%)	1 (0.3%)		
Fever and stomach pain	7 (2.0%)	7 (2.0%)		
Fever and urogenital symptoms	2 (0.6%)	3 (0.9%)		
Fever and suspicion of meningitis	–	2 (0.3%)		
Registration missing	25	40		
(b)				
Triage response—staying at home vs hospital referral	197 (53.1%) triaged to staying at home	213 (55.6%) triaged to staying at home	0.90 (0.68–1.20) video vs. telephone	0.49*
Urgency among hospital referrals—PED versus PUCC	92 (52.9%) referred to PED	50 (29.5%) referred to PED	**2.69** (1.73–4.20) video versus telephone	< 0.001*

Bold numberes denoted the significant findings

*OR* odds ratio; *IQR* interquartile range

*logistic regression; *ref.* reference; *PED* pediatric emergency department; *PUCC* pediatric urgent

The outcome of the patients assessed at hospitals within 8, 24 and 48 h after the calls is shown in Table 2. There was no difference in the total number of patients assessed at hospital at any time. However, children from the video triage group more often received in-hospital treatment, paraclinical testing and/or hospitalization than children from the telephone triage group (Table 2). No adverse events were registered in either group.

**Table 2:** Outcomes of patients assessed at hospital in the video- and telephone triage group respectively, within 8, 24 and 48 h after the call

	Triage group		p value	OR (95% CI)
	Video	Telephone		
0–8 HOURS AFTER THE CALL Patients assessed at hospital, n (%)	175 (47.2%)	173 (45.2%)	0.58*	
Hospital outcome, n (%)			**0.003**^**#**^	
No paraclinical testing/treatment/hospitalization	113 (64.6%)	140 (80.9%)		1.0 (ref.)
Received paraclinical testing/treatment and/or hospitalized < 12 h	48 (27.4%)	26 (15.0%)		**2.29** (1.34–3.92)
Hospitalized ≥ 12 h				
Hospitalized ≥ 12 h	14 (8.0%)	7 (4.1%)		2.48 (0.97–6.35)
Received paraclinical testing	41 (23.6%)	48 (27.8%)	0.37*	
Received treatment	55 (31.6%)	29 (16.8%)	0.001*	
Received prescription	35 (20.1%)	50 (28.9%)	0.06*	
Median temperature, °C (IQR) (n)	38.5 (37.7–39.2) (120)	38.4 (37.6–39.3) (81)	0.8^¤^	
0–24 HOURS AFTER THE CALL Total number of patients assessed at hospital, n (%)	188 (50.7%)	177 (46.2%)	0.22*	
Hospital outcome, n (%)			**0.003**^**#**^	
No paraclinical testing/treatment/hospitalization	121 (64.7%)	143 (80.8%)		1.0 (ref.)
Received paraclinical testing/treatment and/or hospitalized < 12 h	50 (26.7%)	27 (15.3%)		**2.17** (1.28–3.68)
Hospitalized ≥ 12 h	16 (8.6%)	7 (4.0%)		**2.68** (1.07–6.73)
Received paraclinical testing	42 (22.3%)	50 (28.3%)	0.20*	
Received treatment	59 (31.4%)	30 (17.0%)	0.012*	
Received prescription	35 (18.6%)	52 (29.4%)	0.017*	
Median temperature, °C (IQR) (n)	38.4 (37.7–39.2) (127)	38.5 (37.6–39.3) (82)	0.65^¤^	
0–48 HOURS AFTER THE CALL Total number of patients assessed at hospital, n (%)	194 (52.3%)	187 (48.8%)	0.34*	
Hospital outcome, n (%)			**0.005**^**#**^	
No paraclinical testing/treatment/hospitalization	128 (66.0%)	152 (81.3%)		1.0 (ref)
Received treatment/paraclinical testing and/or hospitalized < 12 h	50 (25.8%)	28 (15.0%)		2.0 (ref) **2.12** (1.26–3.56)
Hospitalized ≥ 12 h	16 (8.3%)	7 (3.7%)		**2.55** (1.01–6.43)
Received paraclinical testing	44 (22.7%)	51 (27.3%)	0.27	
Received treatment	59 (30.4%)	30 (16.0%)	0.001*	
Received prescription	35 (18.0%)	53 (28.3%)	0.020*	
Median temperature, °C (IQR) (n)	38.4 (37.7–39.2) (129)	38.5 (37.6–39.3) (88)	0.70^¤^	
Adverse events^	0	0		
Diagnoses, n (%)			**0.001**^**#**^	
Upper respiratory tract infections	47 (24.2%)	60 (32.1%)		0.75 (0.38–1.49)
Obstructive laryngitis	31 (16.0%)	14 (7.4%)		2.12 (0.91–4.97)
Viral infection	24 (12.4%)	23 (12.3%)		1.0 (ref.)
Fever	14 (7.2%)	13 (7.0%)		1.03 (0.40–2.66)
Gastroenteritis	13 (6.7%)	5 (2.7%)		2.49 (0.77–8.10)
Pneumonia	13 (6.7%)	5 (2.7%)		2.30 (0.70–7.56)
Ear infection	12 (6.2%)	33 (17.7%)		**0.35 (0.15–0.84)**
Asthma/bronchitis	10 (5.2%)	6 (3.2%)		1.60 (0.50–5.11)
Other^$^	30 (15.5%)	28 (15.0%)		

Bold numberes denoted the significant findings

*chi-square test

^#^multinomial logistic regression, ref.: reference, IQR: interquartile range

^¤^Mann–Whitney test

^defined as death, signs of lasting means or transfer to intensive care unit

^$^diagnoses with ≤ 5 patients (*observation due to suspected illness, dehydration, influenza, constipation, stomach pain, distortion of finger, febrile seizures, skin symptoms, RS-virus/bronchiolitis, tonsilitis and hospital contact aborted by patient*)

The number of children with different diagnoses varied significantly (p = 0.001), where the usually mild infectious diseases such as ear infection and tonsillitis dominated in the telephone triage group, Table 2. Patients in the video triage group tended to receive potentially more severe diagnoses such as obstructive laryngitis, gastroenteritis, pneumonia, and respiratory syncytial virus-infection.

### Degree-of-worry

The study received 320 parental responses regarding DOW (response rate 42%), Table 3. DOW were not normally distributed, and the results are hence presented with median and IQR. Medan DOW *before* the call was significantly higher in the video triage group (4 vs. 3, p = 0.001). There was a significant association between parental DOW and triage mode, i.e., the call-handler’s choice of triaging the call by video or telephone was affected by the parent’s worry (p = 0.009).

**Table 3:** Degree-of-worry of parents and call-handlers, surveyed after the call by electronic questionnaires

Parents	Video triage group (n = 171)	Telephone triage group (n = 149)	p value^#^
*Degree-of-worry*			
DOW before call, median (IQR)	4.0 (3.0–4.0)	3.0 (3.0–4.0)	**0.001**
DOW after call, median (IQR)	2.0 (2.0–3.0)	2.0 (1.0–3.0)	0.09
Difference between DOW before and after the call in the respective triage group^#^, mean (SD; 95% CI)	1.16 (1.16; 0.99–1.34), **p < 0.001**	1.05 (0.98; 0.89–1.21), **p < 0.001**	0.19

Call-handlers	Video triage group (n = 350)	Telephone triage group (n = 343)	
DOW, median (IQR)	2.0 (1.0–3.0)	2.0 (1.0–2.0)	0.38

Bold numberes denoted the significant findings

Degree-of-worry ranged from 1 (minimal worry) to 5 (maximal worry)

*DOW* degree-of-worry; *SD* standard deviation; *IQR* interquartile range; *95% CI* 95% confidence interval

^#^Kruskal Wallis-test

DOW decreased significantly in both groups after the call (p < 0.001), and DOW *after* the call was no longer significantly different between the two groups.

The call-handlers’ response rate regarding DOW was 92%, and there was no significant difference between the groups, Table 3.

Logistic regression analyses showed that rating DOW as high *before* having talked to a call-handler was significantly associated with assessment at hospital within the next 48 h as compared to low DOW, when studying all parents combined or only the telephone group, Table 4. Medium or high DOW *after* the call were significantly associated to assessment at hospital in both triage groups separately, and when all responses were combined, Table 4.

**Table 4:** Association of parents’ DOW with assessment at hospital within 48 h

	n	OR (95% CI)	p value*
*DOW before call*			
All parents			
Low	51	1.0 (ref.)	0.06
Medium	121	1.25 (0.64–2.43)	
High	148	**1.98 (1.03–3.79)**	
Video triage group			
Low	18	1.0 (ref.)	0.48
Medium	64	1.15 (0.39–3.34)	
High	89	1.61 (0.57–4.52)	
Telephone triage group			
Low	33	1.0 (ref.)	0.05
Medium	57	1.39 (0.58–3.31)	
High	59	**2.78 (1.16–6.70)**	
*DOW after call*			
All parents			
Low	196	1.0 (ref.)	** < 0.001**
Medium	89	**4.63 (2.69–7.95)**	
High	35	**12.09 (4.48–32.60)**	
Video triage group			
Low	96	1.0 (ref.)	** < 0.001**
Medium	58	**4.50 (2.24–9.03)**	
High	17	**19.17 (4.11–89.50)**	
Telephone triage group			
Low	100	1.0 (ref.)	** < 0.001**
Medium	31	**6.80 (2.56–18.08)**	
High	18	**8.16 (2.22–30.04)**	

Bold numberes denoted the significant findings

*DOW* degree-of-worry; *OR* odds ratio; *CI* confidence interval; *ref.* reference

*Logistic regression

## Discussion

Our primary aim was to investigate if video triage could enable more ill children to stay at home, while also efficiently identifying potentially severely ill children with the need for assessment at hospital. There was not a difference in the number of patients triaged to stay at home or in the number of patients assessed at hospital within the follow-up period of 48 h after the call. However, several findings pointed towards video having been used at the discretion of the call-handlers, and not in every other call, as instructed. As such, the video triaged children more often were assessed as potentially more ill by the call-handlers, as they more frequently were referred directly to a PED as opposed to a PUCC reserved for minor illnesses. Moreover, among the children assessed at hospitals, children from the video triage group more often received paraclinical testing or in-hospital treatment and were more often hospitalized as compared to the telephone triage group. Equivalently, the children in the telephone triage group assessed at hospitals received less severe diagnoses, but more prescriptions, probably due to them being assessed in PUCCs with illnesses that could be treated at home with e.g. antibiotics, such as uncomplicated tonsillitis and ear infections.

Our secondary aims related to the parents’ DOW. DOW was higher in the video group than in the telephone group, and there was a significant association between DOW and triage mode, that is, the parents’ degree of worry might have influenced what triage mode that was used. The conversation with the call-handler had in itself a reassuring effect on the parental DOW, as DOW decreased significantly after the call in both groups. Furthermore, high parental DOW both before and after having talked to a call-handler was significantly associated with the child being assessed face-to-face at a hospital. Hence, children of worried parents were actively allocated to video triage. After the call, where the call-handler confirmed the parents’ worry by referring the child to a hospital, the parent had a persisting high worry.

Decision-making is the final product and essential element of call-handling [1, 16]. However, there are several difficulties on the path leading to the most optimal decision. Perceiving the un-spoken, reading, or rather listening, between the lines and using non-verbal communication are crucial capabilities of call-handlers, and not being able to see facial expression or physical movements and being forced to second-hand consultations have repeatedly been reported as the biggest challenges in telephone triage [1–3]. Building a mental picture of the caller using cues gained during the call helps to assess the urgency and thus to make the correct triage decision [16]. Using video triage may assist in the picture-building as some of the aforementioned challenges can be alleviated. As previous results from the study showed an increased reassurance regarding triage decision when using video triage [24], and the current findings indicate that video triage is a compelling choice in cases of more severely ill children, video triage might be a way to increase reassurance of both parents and call-handlers and to optimize triage. This might be especially relevant in cases of complex or potentially severe symptoms and when the parents appear worried. A recent Norwegian study concerning video streaming in emergency calls confirms that the visual input provided by video streaming increases the reassurance of the call-handler [28]. This qualitative study also found that the call-handlers found it easier to decide on the right care and therefore also utilizing resources more wisely.

A systematic review of unscheduled pediatric healthcare visits found that patients do not find these visits as clearly distinguished from planned visits as professionals, and parents do not necessarily take the appropriateness of visits into account, when choosing where and when to seek help [29]. Therefore, it is important to focus on helping parents navigating the healthcare system and improve challenges in the system of unscheduled health care. One method could be video triage at OOH call-centers, as the number of hospital visits and contacts to OOH centers potentially can be decreased, both by optimizing the triage and by reassuring parents so that more can care for their children at home without need for renewed contact to the health care sector. This study group previously reported that parents felt more reassured after video triage and were also relieved that they in some cases could stay at home rather than having to go to the hospital, as a health care professional already had “seen” the child [24].

Several studies have tried to estimate appropriateness of referrals made by call-centers by assessing patient outcome [30–32]. Appropriateness has been judged either by the evaluating hospital physician or retrospectively by physicians studying patient charts. The number of under-referrals has been estimated by how many patients that were initially recommended home care or assessment by a GP but later got assessed at a hospital anyhow [30–32]. Naturally, some of these cases may be caused by normally occurring deterioration and is not a result of a sub-optimal triage per se. Largely, call-centers perform well [30, 31], and save money as opposed to self-referrals [33]. Some studies found room for improvement, especially in certain patient categories [30, 34]. However, there is now a tendency towards trying to define appropriateness of triage differently. Instead, call-handling should be patient-centered and involve shared decision-making, where callers are included, guided and supported in their decision-making and thus in achieving empowerment [29, 35]. High parental DOW was associated with the child subsequently being assessed at hospital, corresponding to previous findings [9, 10].

Feverish children are highly prevalent in OOH care, and challenging to assess, as harmless and severe causes may be hard to separate. Furthermore, parents often expect a physical examination. Video triage might be beneficial, as it provides visual assessment and can assist in finding the most severely ill children. Several parents stated that it was a relief not having to go to a hospital after video triage as somebody already had “seen their child” [24]. In two percent of video triage calls, the video streaming did not succeed and in another two percent, the approached parent did not wish to participate. Furthermore, no adverse events were identified. Therefore, video streaming of febrile children was feasible and as safe as telephone triage at this medical helpline, and can be considered for implementation in other call-centers as well.

Video triage in other symptoms and age groups should be the subject of future studies, both to identify in which cases that video triage is most useful, and also how to implement the video findings most optimally in the decision-making protocols used at many call-centers. Randomized studies are needed to find the impact of video triage on triage outcome and subsequent patient outcome.

By including DOW, a simple and time-efficient score, in the triage process, the incorporation of parental feelings of worry in the decision-making can be ensured. As worry is a main reason to OOH contact, this is crucial. By investigating how DOW has changed at the end of the call, it is possible to conclude if the contact has resulted in increased reassurance and empowerment. If not, the underlying reasons should be sought corrected.

Some limitations to the study were identified. Although the participating call-handlers had been instructed to include patients to the video- or telephone triage group in a 1:1 ratio to get two comparable groups, the results show that children with potentially more severe or complex symptoms and/or with parents displaying a high degree of worry more often received video triage than telephone triage. Previously, the study group reported that several of the participating study call-handlers stated that they could not do without video in some situations, and therefore switched to video triage even if the allocation strategy stated telephone triage [24]. Some call-handlers also reported to find it easier to reassure parents when using video. Therefore, it is possible that the more worried the parents seemed to be or the more ill the child seemed, the more often call-handlers chose to use video instead of telephone triage. Had the groups been more alike, it is possible that an impact of video triage on patient- and triage outcome would have been detectable. The call-handlers previously stated that they often could advise patients to stay at home when using video triage [24]. However, this effect might have gotten counteracted if a large proportion of the video triaged patients were more ill and therefore needed to be referred to a hospital. These findings complicate interpretation of the results. As the allocation strategy was not maintained, confounding by severity has likely affected the results. But on the other hand, as this new tool of video triage seems to have been used in a pragmatically and clinically helpful way, rather than in the way the research team planned, we now know that video triage is useful in assessing complex and/or severe symptoms. This may increase the reassurance felt by the call-handlers concerning triage outcome.

Non-Danish and non-English speakers were excluded, to test this new tool in a setting that felt achievable to both call-handlers and parents. We also did not want to affect the waiting times in a negative way, as might have been the case if there would have been difficulties explaining the video triage setup and tool. For future purposes though, these situations should be studied as well, as it is possible that much is to gain when using a visual aid when experiencing language barriers.

Regarding DOW, parents were asked AFTER the call about their DOW both before and after having talked to a call-handler. Recall bias might have an impact on the “before-rating”. Also, as the numbers of parents rating their DOW as low (1 + 2) were rather small, this generated some wide 95% CI’s.

Lastly, it would have been preferable if the study had been conducted as a randomized controlled trial, but this was not possible due to several reasons. Firstly, the call-handlers did not have sufficient time for thoroughly informing the parents and obtaining written informed consent, and an oral consent, although it would have been stored in the MH1813 electronic patient chart, would not legally suffice. Furthermore, it was not possible to change the computer set-up to randomly allocate calls to video or telephone triage, or to change the introduction speech of MH1813 as would have been desired in terms of informing about the study. Due to this limitation, the study cannot yield fully valid answers to the impact of video triage on triage and patient outcomes.

Finally, to sum the findings up, the study did not find an increase of video triaged patients staying at home. Video triage of young children with fever was feasible, acceptable and as safe at telephone triage at this medical helpline. However, video triage was more often used in situations with complex or potentially severe symptoms and when parents rated worry as high. High parental degree-of-worry was associated to assessment at hospital and can be used as a method to incorporate the caller’s subjective notion of worry and urgency into the triage process.

## Data Availability

The datasets used during the current study are available from the corresponding author on reasonable request.

## Abbreviations

DOW: Degree-of-worry
GP: General practitioner
ICD-10: International Classification of Diseases version 10
MH1813: Medical helpline 1813
OOH: Out-of-hours
PED: Pediatric emergency department
PUCC: Pediatric urgency care clinic
